# Therapeutics of Chronic Constipation

**Published:** 1871-02

**Authors:** 


					﻿THERAPEUTICS OF CHRONIC CONSTIPATION.
Dr. J. K. Splender, in a sensible paper in the Medical Times
and Gazette, on the treatment of constipation, when it exists per
se as a primary disorder of function, recommends the following
judicious plan, which comprises four factors : (a) Minute and
frequent doses of watery extract of aloes, not exceeding one gr.
—very rarely, of extract of colocynth. (Z>) A dose of sulphate
of iron; gr. jss. to gr. ij., always combined with the aperient,
(c) Regulation of the diet, (c?) Constitutional exercise, lie does
not believe that any other drug can supply the place of the sul-
phate of iron, though belladonna may be occasionally useful as an
auxiliary. At first he gives a pill, composed as above, three
times a day, immediately after the principal meals, and gradually
reduces the number and frequency of the pills as the intestinal
functions are restored. The action is slow, requiring two or per-
haps three days, but nothing approaching to purgation ought
ever to be permitted. As a substitute in certain cases, Mist, ferri
co., with dec. aloes co., in corresponding doses, may be adopted.
—Medical and Surgical Reporter.
SULPHITE OF SODA.
Dr. G. Kelmer, of Illinois, writes to the Western Journal of
Medicine, that sulphite of soda can be relied upon as a speedy
and effectual remedy in almost all parasitic affections of the skin.
“ I have also used it in various other cutaneous diseases with
uniform and unvarying success. And in that condition of the
blood which is manifested by the production of numerous furun-
cles, commonly known as boils, the administration of sulphite of
soda, with carminative tonics, has proved, under my observation,
a perfect and rapid remedy. In carbuncles, I know that, after
using the following, it may be relied upon. After a forced or
spontaneous opening of the carbuncle, apply a solution, on lint,
of, say :
R—Sodac sulphitis, .	.	.	? ss.
Acidi carbolici (crys.)	.	.	3ss*
Glycerine,	.	.	. f. 3 iij. M.
“ It is remarkable how rapidly, under these applications, the
ordinarily slow separation of the necrosed cellular tissues takes
place—the destructive process ceases, and healthy granulations
spring up.
“ In uticaria it has proven very successful in subduing the
worst forms of this disease in twenty-four hours. There are a
great many other skin diseases in which I have found it equally
efficient.
“ Another application of this salt, which I consider valuable, is
in the case of infants, by whom their food (the mother’s breast-
milk) is often ejected. A dose of two to five grains of sulphite,
in combination with comp. tr. cardamom, sweetened, has proved
successful in causing a retention and assimilation of the contents
of the stomach when administered soon after imbibition, thus
greatly promoting the health of the child. Also in cases of chil-
dren where there is a fermented, swollen condition of the bow-
els, especially if constipated, the sulphite of soda will remove the
difficulty in a short time.”—Drug Circular and Chemical Gazette.
				

